# Ternary complexes in protein–DNA interactions: Kinetics and mechanisms

**DOI:** 10.1016/j.jbc.2025.111024

**Published:** 2025-12-08

**Authors:** Adina Hefetz, Elena Rogoulenko, Yaakov Levy

**Affiliations:** Department of Chemical and Structural Biology, Weizmann Institute of Science, Rehovot, Israel

**Keywords:** binding kinetics, coarse-grained simulations, protein-DNA recognition, sliding, Sox2 and Oct1 proteins, SRF and SAP1 proteins, ternary complexes

## Abstract

Transcription factors (TFs) play a central role in gene regulation by binding specific DNA sequences. Although extensive research has uncovered various mechanisms governing binary TF·DNA complexes, relatively few structural and mechanistic studies exist for ternary complexes or larger assemblies. This study employs coarse-grained molecular dynamics simulations to investigate how DNA conformation, TF properties, and protein–protein interactions influence ternary complex formation. We focus on 2 TF·TF·DNA systems: Sox2·octamer transcription factor 1 (Oct1)·DNA and SRF·SAP1·DNA, each involving 2 TFs, one of which binds to a bent DNA segment and the other to a more linear DNA region. Our findings reveal that, in both systems, the kinetics of ternary complex formation is primarily governed by nonspecific TF–DNA interactions. Additionally, we find that the DNA conformational change does not always dominate the kinetics, suggesting that a complex interplay of biophysical factors influence ternary complex formation. When the TFs exhibit divergent nonspecific affinities, as in the Sox2·Oct1·DNA system, the binding order is dictated largely by diffusion along nonspecific DNA and stabilized through protein–protein interactions. In contrast, when both TFs display comparable nonspecific affinities, as observed in the SRF·SAP1·DNA system, the binding order becomes more dependent on site-specific recognition and, consequently, on the underlying DNA conformation. This distinction enables the emergence of different mechanisms depending on the structural state of the DNA. This study highlights how the interplay between TF molecular properties, DNA sequence and deformability, and protein–protein interactions together shape the kinetics and molecular mechanisms underlying ternary TF·TF·DNA complex formation.

Transcription, being the process of converting DNA into RNA, is a vital step in gene expression. It involves the assembly of a protein·DNA complex, which is initiated by the binding of RNA polymerase, transcription factors (TFs) that bind to a promoter region of a gene, and other cofactors (1). The role of TFs is to regulate gene expression by binding to specific DNA sequences. By controlling the activation and repression of genes, TFs ensure that genes are transcribed only in the appropriate cells at the right time and in the right amount, thus regulating gene expression (2, 3).

Before initiating the transcription process by binding to their cognate (*i*.*e*.*,* promoter) site on DNA, TFs must first find that site, which they achieve by undertaking an extensive search through a large fraction of the genome (4, 5, 6, 7, 8). This search can be very time-consuming and is affected by environmental conditions (*e*.*g*., salt concentrations (9, 10, 11) or crowding conditions (12, 13, 14, 15, 16, 17) in the cell) and by various molecular properties of the TF (*e*.*g*., its oligomeric state (18), charge density (19, 20), and the presence of disordered regions (21, 22)). The transition from the search mode, when the protein scans the DNA to locate its target site, to the binding mode, when the protein establishes stable sequence-specific interactions at that site, is distinguished by a shift from nonspecific binding, dominated by electrostatic interactions, to specific binding, which involves short-range interactions (2, 23). This transition may depend on several factors. The involvement of conformational changes affecting either the TF or the DNA may increase the energetic barrier for specific binding and thus slow the kinetics for promoter binding. It was shown that molecular frustration between the non-specific and specific binding patches on the TF can modulate the binding kinetics (24, 25, 26). Furthermore, the binding kinetics can be slowed by the occurrence of unproductive binding events in which the TF skips over the promoter, which may prompt additional search rounds until the promoter is found again and specific binding is achieved (27, 28). The frequency of skipping events may depend on the molecular properties of the TF, the degree of conformational changes, and the environmental conditions.

Structural analyses of protein·DNA complexes, obtained through methods such as crystallography, have provided valuable insights into how TFs recognize their specific DNA binding sequences (3). Although extensive research has revealed various recognition mechanisms for binary TF·DNA complexes (such as monomeric or homodimeric TF binding), relatively few structures and mechanisms are known for ternary complexes or larger assemblies, mainly because of the experimental challenges involved in studying the kinetics and thermodynamics of such complex systems (29).

Understanding these complex molecular assemblies is particularly crucial for comprehending gene regulation in higher organisms (*e*.*g*., mammals, among them humans), where transcriptional control requires more sophisticated mechanisms (30, 31) (such as cooperative interactions among multiple TFs (32, 33, 34, 35)) to enable precise genomic targeting and complex information processing at the individual regulatory element level (36, 37).

In this context, DNA shape may play a pivotal role, not only in gene regulation but also in genome organization and in the maintenance of genomic integrity (38, 39). It has been proposed that proteins not only recognize, but also exploit, the intrinsic flexibility of DNA to undertake their function (40, 41, 42, 43, 44, 45). Adopting a specific DNA conformation that deviates from its standard structure requires energy to overcome the deformation energy barrier. This highlights the critical role of DNA flexibility in facilitating transcriptional complex assembly (46). Overall, DNA conformation is essential for gene regulation and combinatorial control, and nature has evolved strategies to overcome the deformation energy barrier (47, 48, 49).

In this study, we aim to investigate inter-TF cooperativity as a mechanism by which large protein·DNA assemblies can overcome the DNA-deformation energy barrier and thus form ternary TF·TF·DNA complexes even when DNA bending makes their formation energetically unfavorable. We propose that a pre-bound TF can assist a partner protein bind to a bent DNA site. To explore this hypothesis, we analyzed the kinetics and mechanisms of ternary complex formation across various DNA conformations using coarse-grained molecular dynamics (CG-MD) simulations. To examine the impact of DNA deformability on complex formation, specifically, we employed a deformable DNA model developed in our group (27). This model, adapted for CG-MD simulations, is optimally suited to the study of complex biological systems. Uniquely, it incorporates the ability to transition between linear and bent DNA conformations, providing a realistic representation of how different DNA sequences exhibit distinct dynamics behaviors over time in the context of TF interactions.

Our study focuses on two ternary complex systems, each involving 2 TFs, one of which binds to a bent DNA region (Fig. 1). The first ternary complex is between Oct and Sox TFs and DNA (Sox2· octamer transcription factor 1 (Oct1)·DNA; PDB ID: 1O4X), with this complex known to play key roles in neural development and embryogenesis (50). The Oct1 and Sox2 proteins often bind adjacent DNA sites and interact through their DNA-binding domains to regulate transcription synergistically (29). The second ternary complex studied here is between the serum response factor (SRF) and SAP1 TFs and DNA (SRF·SAP1·DNA; PDB ID: 1HBX). In the SRF·SAP1·DNA system, the DNA sequence is critical for the rapid transcriptional activation of the human c-fos protein, which is a proto-oncogene expressed in response to external cellular cues (51). Full activation of the serum response element (SRE) in this system requires cooperative binding of SRF to its bent DNA-binding site and of SAP1 to its specific site (52).

**Figure 1:**
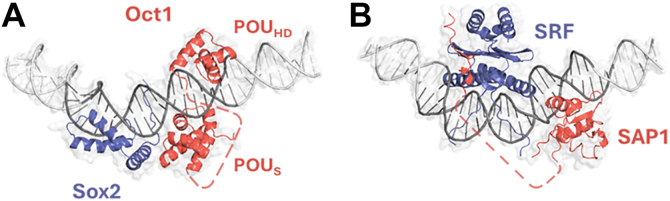
**Structures of the two studied ternary complex systems formed between transcription factors and DNA**. *A*, ternary Sox2·Oct1·DNA complex–A ternary complex consisting of the transcription factors Sox2 sex determining region Y-box 2; *blue*) and Oct1 (octamer transcription factor 1; *red*) bound to DNA (*gray*). Sox2 binds in the minor groove of the DNA, introducing a ∼50° bend, and also interacts with Oct1’s POU-specific (POU_S_) domain, while Oct1’s POU homeodomain (POU_HD_) binds the DNA from the opposite side. The structure was determined using solution NMR (pdbID: 1O4X). *B*, ternary SRF·SAP1·DNA complex–A ternary complex involving the SRF homodimer transcription factor (serum response factor; *blue*) and the SAP1 ternary complex factor (SRF accessory protein 1; *red*) bound to DNA (*gray*). SRF binds to the minor groove of the DNA, disrupting its structural integrity and inducing a bend of 77°. This structure was resolved *via* X-ray diffraction (pdbID: 1HBX). The *red dashed lines* represent disordered linkers connecting the POU_S_ and POU_HD_ domains of Oct1 (panel *A*; linker length 26 residues) and connecting the B-box to the core SAP1 domain (panel *B*; linker length 43 residues). These regions were not resolved in the experimental structures and were reconstructed using the Modeller program. The experimentally-resolved DNA (*dark gray*) was extended for both ternary complexes to 100 base pairs (*light gray*). Oct1, octamer transcription factor 1.

By leveraging CG-MD simulations, this research investigates the mechanisms governing ternary TF·TF·DNA complex formation, focusing on the effects of DNA conformation, TF properties, and protein–protein interactions on binding kinetics and order, with the aim of addressing fundamental biophysical questions about ternary complex assembly processes. We focus on cases where the 2 TFs diffuse independently along the DNA, rather than on a TF dimer that binds DNA as a preformed complex (53, 54). Our goal is to determine how these factors influence the assembly process and contribute to cooperative binding in transcriptional regulation. To achieve this goal, we selected for analysis two model ternary systems that have been studied experimentally, providing molecular insights into their formation mechanisms, where each system consists of two transcription factors with a defined protein–protein interface and in which 1 TF binds to a bent DNA region. Such characterization of the molecular mechanisms may contribute to a better understanding of gene regulation and the interplay between DNA structure, TF activity, and cooperative interactions in transcriptional control.

## Results

### Kinetics of ternary complex formation

#### Formation kinetics of the Sox2⋅Oct1⋅DNA ternary complex

To understand the kinetics of complex formation between Oct1, Sox2, and DNA, we studied the formation kinetics of their complexes by measuring the association rate (k_on_) constants. In order to examine how the ability of DNA to deform affects the kinetic parameters, we used a deformable DNA model in which the energetic preference to DNA deformation is controlled by a single parameter *α* that is further described in supporting information. At high α values the DNA backbone has a high energetic preference towards the deformed conformation, and at low values of α the canonical B-DNA structure is energetically preferred. We first examined k_on_ values for binary complexes in which either Oct1 or Sox2 binds to its cognate DNA site and then investigated k_on_ for ternary complexes in which Oct1 binds to a preformed Sox2⋅DNA complex and in which Sox2 binds to a preformed Oct1⋅DNA complex. Both the binary and ternary association rates are valuable when describing the molecular mechanism of formation of this ternary complex. By analyzing the effect of the pre-binding of one protein on the association rate of the other protein, we may observe cooperativity between them. Such stepwise binding *via* cooperativity between the two proteins to form the ternary complex may affect their search dynamics and therefore the binding kinetics and mechanism.

**Figure 2:**
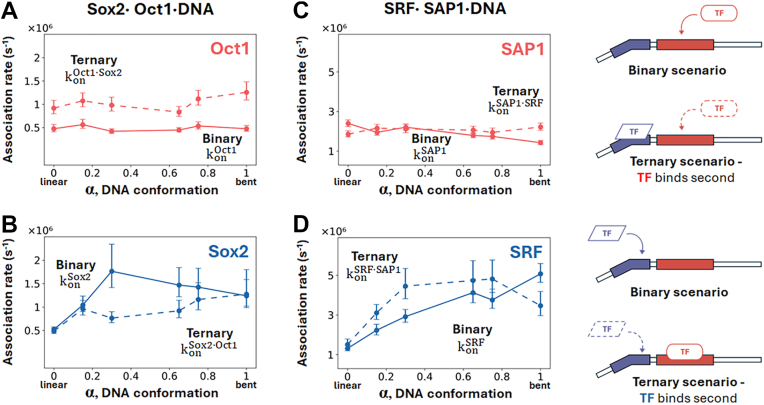
**Kinetics of TF·TF·DNA ternary complex formation as DNA conformation transitions from linear to bent**. Association rates are shown for binary (*solid lines*) and ternary (*dashed lines*) complexes as a function of DNA conformation (α) for two ternary protein·DNA complexes: the Sox2·Oct1·DNA system (*A* and *B*) and the SRF·SAP1·DNA system (*C* and *D*). In each panel, the binding of the specified TF to DNA to form the binary complex (*solid line*) is compared with its binding to preformed TF·DNA to form the ternary complex (*dashed line*) as the DNA conformation changes from linear to bent. *A*, association rates of Oct1 binding to DNA alone ($k_{on}^{Oct1}\text{;}$ to form the binary complex) and of Oct1 binding to DNA pre-bound with Sox2 (*i*.*e*.*,* of Oct1 binding to pre-bound Sox2·DNA, $k_{on}^{Oct1\cdot Sox2}\text{;}$ to form the ternary complex), where Oct1 binding rates refer to the binding of the POU_S_ domain. *B*, association rates of Sox2 binding to DNA alone ($k_{on}^{Sox2}$; to form the binary complex) and to pre-bound Oct1·DNA ($k_{on}^{Sox2\cdot Oct1}\text{;}$ to form the ternary complex). *C*, Association rates of SAP1 binding to DNA alone ($k_{on}^{SAP1}$; binary) and to pre-bound SRF·DNA ($k_{on}^{SAP1\cdot SRF}\text{;}$ ternary). *D*, association rates of SRF binding to DNA alone ($k_{on}^{SRF}$; binary) and to pre-bound SAP1·DNA ($k_{on}^{SRF\cdot SAP1}$, ternary). *Error bars* indicate SEM from multiple binding events. Each rate was estimated from at least 100 first passage times. The schemes on the right show two transcription factors (TFs) binding to their respective adjacent cognate sites on DNA (*solid blue* and *red shapes* on the *black tube* representing DNA). Binary scenarios involve binding by either the *red* TF to its cognate site (first scheme) or the *blue* TF to its cognate site (third scheme), whereas in the ternary scenarios, a freely diffusing TF (shown with *dashed lin*e borders) binds a prebound TF·DNA binary complex. TF, transcription factor.

**Figure 3:**
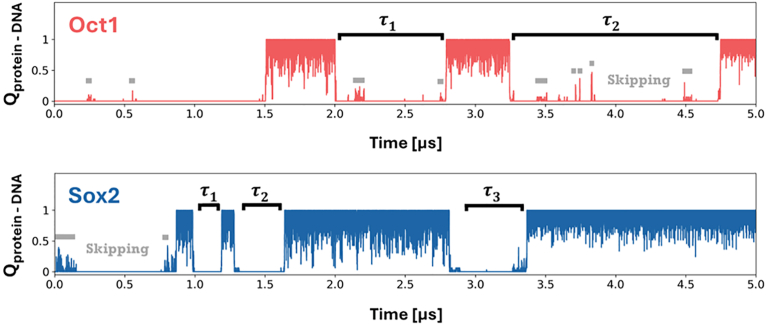
**Representative trajectories of binary TF–DNA binding for Oct1 (*top*, *red*) and Sox2 (*bottom*, *blue*) interacting with mostly-bent DNA (α = 0.65)**. Each trajectory shows the time evolution of the fraction of native contacts between the TF and its target DNA site (Q_Protein-DNA_), thus capturing transitions between binding states. Periods marked as τ represent representative association times, defined as the duration of the transition from the search mode (Q_Protein-DNA_ = 0) to the recognition mode (Q_Protein-DNA_ > 0.65). *Gray horizontal bars* indicate skipping events in which partial contacts form but the TF dissociates before achieving the fully-bound recognition state, resulting in an unproductive binding attempt. TF, transcription factor.

The binary binding rate constant for Oct1 binding with DNA differs considerably from that of the Sox2 protein binding with DNA. Sox2 exhibits higher binary association rates than Oct1 across all examined DNA conformations, indicating that Sox2 has faster kinetics for binding its target site. The difference between the binary complex formation rates of Oct1 (Fig. 2*A*, solid line) and Sox2 (Fig. 2*B*, solid line) increases as the DNA conformation becomes more bent, with Sox2 forming a binary complex with DNA three-fold faster compared with formation of the analogous complex between Oct1 and DNA. Representative trajectories for Oct1 and Sox2 at α = 0.65 (Fig. 3), when the DNA is considerably prone to deformation, further illustrate these contrasting binary binding rates. Oct1 undertakes more skipping events (horizontal gray lines) than Sox2, whereas Sox2 moves out of search mode (Q_Protein-DNA_ = 0) and into recognition mode (Q_Protein-DNA_ > 0.65) faster, more frequently, and for longer than Oct1. Consequently, Sox2 forms binary complexes more rapidly and repeatedly, reflecting its faster binding kinetics. The presence of Sox2 at its binding site significantly enhances the binding kinetics of Oct1, as shown by the ternary association rates being approximately double the binary association rates regardless of DNA conformation (Fig. 2*A*). DNA conformation does not significantly affect either the binary or ternary association rates of Oct1 (Fig. 2*A*), which is expected given that the Oct1 binding site resides on a linear DNA segment. However, for the Sox2 protein (Fig. 2*B*), whose cognate binding site is characterized by a bent DNA conformation, the association rate shows a clear dependency on DNA conformation. As the DNA becomes more bent (*i*.*e*., at high *α* values), the association rate of the Sox2 protein increases for the formation of both binary and ternary complexes. In contrast to the effect of pre-bound Sox2 protein on the binding of Oct1 protein to DNA (Fig. 2*A*, dotted line), the effect of pre-bound Oct1 protein on the binding of Sox2 is negligible (Fig. 2*B*, dotted line), that is, when Oct1 occupies its cognate DNA binding site, the association rate of Sox2 to its DNA binding site is hardly affected. This is illustrated by a ∼1:1 ratio of ternary:binary association rates at the extremes (linear vs bent) but not so much for intermediate alpha values (α ≈ 0.3–0.7).

**Figure 4:**
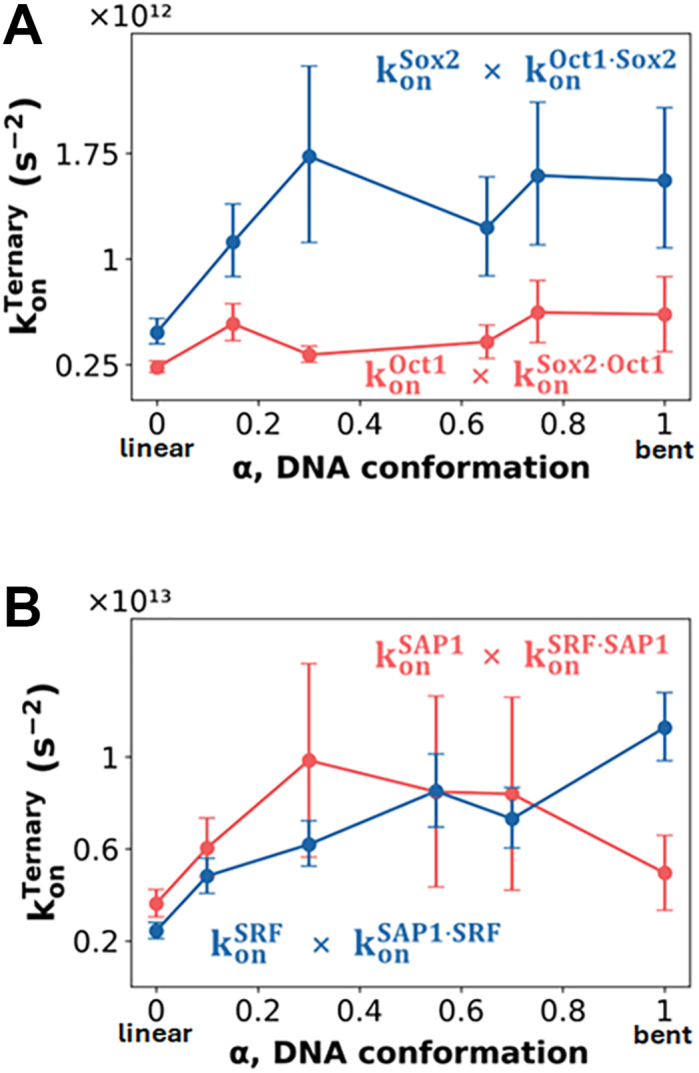
**Overall rate of ternary complex formation for distinct binding mechanisms as a function of DNA conformation (α)**. *A*, Sox2·Oct1·DNA system: The *red curve* represents the total rate of ternary association when Oct1 binds first, followed by Sox2 ($k_{on}^{Oct1}\;\cdot \;k_{on}^{Sox2\cdot Oct1}$). The *blue curve* represents the reverse binding order, with Sox2 binding first, followed by Oct1 ($k_{on}^{Sox2}\;\cdot \;k_{on}^{Oct1\cdot Sox2}$). *B*, SRF·SAP1·DNA system: The *red curve* corresponds to SAP1 binding first, followed by SRF ($k_{on}^{SAP1}\;\cdot \;k_{on}^{SRF\cdot SAP1}$), whereas the blue curve represents SRF binding first, followed by SAP1 ($k_{on}^{SRF}\;\cdot \;k_{on}^{SAP1\cdot SRF}$). *Error bars* indicate SEM from multiple binding events. Each rate was estimated from at least 100 first passage times.

We estimated the overall rates of ternary complex formation for the two binding scenarios, that is, where Sox2-binding precedes Oct1-binding and *vice versa*. This analysis showed that the fastest pathway to form the ternary complex involves Sox2 binding the DNA first, followed by Oct1, a trend that remains consistent across all DNA conformations (Fig. 4*A*). When Sox2 binds first, the increased rate of ternary complex formation is driven primarily by the influence of DNA conformation on Sox2's binding rate. In contrast, the binding rate of Oct1, which represents the final step of ternary complex formation, is unaffected by DNA conformation. This suggests that, whereas DNA conformation significantly impacts Sox2 binding, it has no effect on Oct1 binding. Instead, the kinetics of Oct1 binding is modulated by Sox2 (see also Fig. S5).

**Figure 5:**
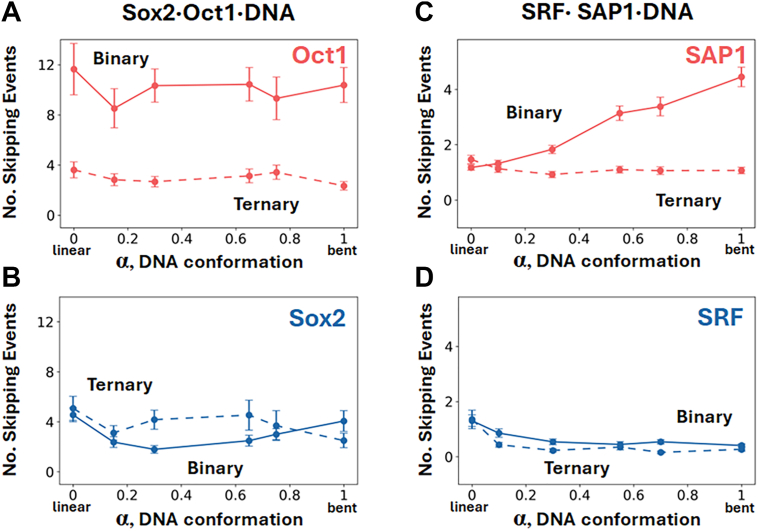
**Number of unproductive binding events**. Number of skipping events during the formation of binary TF·DNA complexes (*solid line*) and ternary TF·TF·DNA (*dashed line*) complexes as a function of DNA conformation (α). Skipping events as: *A*, Oct1 binds to DNA or to a preformed Sox2·DNA complex; *B*, Sox2 binds to DNA or to a preformed Oct1·DNA complex; *C*, SAP1 binds to DNA or to a preformed SRF·DNA complex; and (*D*) SRF binds to DNA or to a preformed SAP1·DNA complex. *Error bars* indicate SEM from multiple binding events. Each rate was estimated from at least 100 first passage times. TF, transcription factor.

To investigate molecular factors that may explain the differences between the binding kinetics of Sox2 and Oct1 to DNA, as well as the modulatory effect of Sox2 on Oct1 binding, we calculated the occurrence of unproductive binding events in which the protein was located at the binding site but skipped before complete binding took place. The number of such skipping events was estimated from the CG-MD simulations for the formation of either binary or ternary complexes of the two proteins and for various DNA conformations. In the context of searching DNA to form a binary complex, Sox2 exhibits significantly fewer skipping events compared with Oct1 (Fig. 5*B*), a trend that correlates with its faster binary binding kinetics (see Fig. 2, which shows shorter search-to-recognition mode transition times and fewer unproductive binding attempts for Sox2–DNA compared with Oct1–DNA interactions). Skipping events by Oct1 in the ternary scenario are significantly reduced compared with the binary scenario, indicating that pre-bound Sox2 substantially decreases the number of Oct1 skipping events (Fig. 5*A*). This may explain how pre-bound Sox2 enhances the rate of ternary complex formation by Oct1 compared with the kinetics of formation of its binary complex. In contrast, pre-binding of Oct1 at its cognate DNA site minimally impacts the number of skipping events by the Sox2 protein compared with its binding in the absence of the Oct1 protein (Fig. 5*B*, binary *c*.*f*. ternary complex formation). The observed trends for both binary and ternary complexes support a mechanism in which Sox2 binds first, and subsequently modulates Oct1 binding kinetics by reducing skipping events and facilitating DNA binding through protein–protein interactions.

Contrary to our initial hypothesis, the kinetic analysis revealed that DNA conformation is not necessarily the rate-determining factor. Rather, Sox2 (which binds a bent DNA region) consistently binds first and facilitates the subsequent binding of Oct1 (even though it binds a straight DNA region), rather than these TFs binding in reverse order. This trend is observed across all examined DNA conformations, both linear and bent (Fig. 4*A*).

### Formation kinetics of the SRF⋅SAP1⋅DNA ternary complex

Similarly to the ternary complex formed between Oct1, Sox2, and DNA, the kinetics of the formation of a ternary complex between the SRF and SAP1 proteins and DNA was studied by CG-MD simulations by comparing the formation rates for the binary and ternary complexes. Figure 2 compares for each of the proteins the association rates of binary complex and of ternary complex, where the other protein is pre-bound at its DNA cognate site. For SAP1, the association rate for binary complex formation with DNA shows a small dependency on DNA conformation. For the ternary scenario, when SRF is pre-bound at its DNA cognate site, the association of SAP1 is assisted by the presence of the SRF protein primarily for bent DNA conformations (Fig. 2*C*). Comparing between the rate constants for SAP1 binding to form the binary and ternary complexes suggests a shift in the role of SRF recruitment, with its contribution becoming more apparent as DNA bends. SRF enhancement of SAP1 binding to bent DNA conformations is illustrated by the ratio between the ternary and binary rate constants, which is around one for linear DNA but reaches a value of 1.6 for bent DNA. Figure 2*D* focusses on SRF-binding, comparing the association rates for formation of the binary SRF·DNA complex with the corresponding rates for the binding of SRF to preformed SAP1·DNA to form the ternary complex. The binary complex formation rate shows a strong dependence on DNA conformation. SRF binding to a bent DNA is about 5-fold faster than to a linear B-DNA conformation, which is consistent with SRF binding to a bent region, as shown in the crystal structure of the complex (Fig. 1*B*). This trend for binary SRF·DNA complex formation is the opposite of that found for binary SAP1·DNA complex formation, in which the rate decreases as DNA becomes more bent. The kinetic rate for ternary complex formation (*i*.*e*., when SRF binds to prebound SAP1·DNA) is often greater than the kinetic rate for binary complex formation, with the exception of highly bent DNA, where the binary rate is greater than the ternary rate. This suggests that SAP1 assists the binding of SRF for DNA conformations in which the cognate site is not bent. Indeed, the ratio between ternary and binary rates reaches a value of 1.5 for various DNA conformations, but drops to 0.7 for the bent DNA conformation. We note that the ratio between ternary and binary rates approaches a value of 2.5 for the Oct1⋅Sox2⋅DNA system, whereas for the SRF⋅SAP1⋅DNA system the maximal value is 1.5, reflecting greater cooperativity in the former system.

We sought to determine whether the combined effects of the association rate trends influence the preferred mechanism for ternary complex formation by SRF and SAP1 with DNA. To address this, we calculated the rate of ternary complex formation for both binding scenarios across various DNA conformations. Figure 4*B* shows that the ternary kinetics of the two binding scenarios (SRF-first *versus* SAP1-first) are broadly similar to each other for most DNA conformations. Consequently, a distinct preference for a specific TF binding order across DNA conformations is not observed for SRF⋅SAP1⋅DNA, unlike the ternary complex of Oct1⋅Sox2⋅DNA, which shows a clear preference for Sox2-first binding, irrespective of DNA conformation. Nevertheless, a clear shift in preference between the two complex formation mechanisms is observed for SRF⋅SAP1⋅DNA as it becomes more bent. Whereas, at more-linear DNA conformations (up to α = 0.3), faster complex formation is obtained when the SAP1 protein binds first, as the DNA becomes more bent, the preference shifts and the ternary complex is formed faster when SRF binds first, with this preference most pronounced for highly bent DNA. This analysis suggests that, as DNA becomes more bent, the association rate for SRF·DNA binary complex formation increases significantly, leading SRF-first binding to be favored for ternary complex formation (*i*.*e*.*,* favoring formation of SRF·DNA followed by SAP1 binding to form the ternary complex). For less bent DNA, the opposite scenario, in which SAP1 binds prior to SRF binding, is slightly favored. DNA conformation therefore appears to play a central role in modulating these rates and, consequently, the underlying mechanisms of ternary SRF⋅SAP1⋅DNA complex formation (Figs. 4*B* and S5).

To explore whether skipping events can explain the association rate trends from a binding kinetics perspective, we analyzed the number of skipping events in the binary and ternary processes for SAP1 and SRF binding with DNA. For SAP1, the presence of prebound SRF reduces the number of skipping events to an average of approximately a single event across all DNA conformations (Fig. 5*C*, dashed line), which aligns with the observed similar association rates across DNA conformations (Fig. 2*C*, dashed line). In the binary SAP1·DNA complex, the number of skipping events increases as DNA bending intensifies (Fig. 5*C*, solid line), correlating with a reduction in the binary association rate (Fig. 2*C*, solid line). For SRF binding, skipping events occur in low numbers for the formation of both binary and ternary complexes (Fig. 5*D*). The mean number of skipping events is greater for the binary case than the ternary case, which indicates that pre-bound SAP1 reduces the number of skipping events by SRF. Skipping events approach zero when SRF binds bent DNA (Fig. 5*D*). The SRF-binding trends observed for the formation of both binary and ternary complexes correspond to the association rates shown in Figure 2*D*, where higher binding rates can be explained by a decrease in skipping events until full binding is complete. However, since the mean number of skipping events is already low, this effect is not particularly strong and can be attributed primarily to the local electronegative potential of the bent DNA. The reduction in skipping events during SAP1 binding in the presence of pre-bound SRF may also be influenced by this local electronegative potential. Bent DNA offers favorable nonspecific electrostatic interactions compared with linear DNA, however, in the presence of SRF, the SAP1 protein loses the option of binding bent DNA, which reduces the likelihood of skipping events from the cognate site of SAP1 to the bent DNA region.

### DNA search by the transcription proteins prior to formation of the ternary TF⋅TF⋅DNA complex

Another molecular factor that may explain differences in the binding kinetics of various TFs is their diffusion properties along non-specific DNA, as the kinetics of their search for their DNA cognate site can influence the association rate for both binary and ternary complex formation. Accordingly, the nature of TF interactions with nonspecific DNA sites can affect the kinetics of binding to the specific DNA sites. The differences in association rates can therefore be better understood by quantifying the nature of linear diffusion along non-specific DNA.

Linear diffusion of proteins along DNA is governed by electrostatic interactions (55) and was studied at two salt concentrations, 0.02 M and 0.04 M, for the four proteins that participate in the two ternary complexes with DNA. To quantify this motion, we calculated the mean square displacement (MSD) of the center of mass of each protein from a static DNA aligned to a Z axis, from which we calculated the corresponding linear diffusion coefficients.

**Figure 6:**
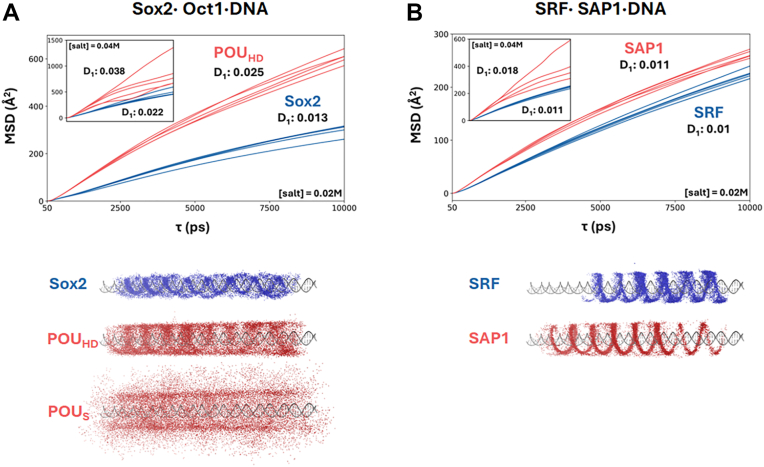
**Analysis of TF diffusion dynamics along non-specific DNA**. *A*, one-dimensional diffusion coefficient (D_1_) calculations based on MSD profiles for five trajectories of Sox2 (*blue*) and the POU_HD_ domain of Oct1 (*red*) at 0.02 M and 0.04 M salt concentrations. Only proteins exhibiting ≥70% linear diffusion (sliding + hopping) were included in the analysis. The values of D_1_ Å^2^ ps^-1^ were derived from the MSD between 50 ps and 10 ns. Standard errors for POU_HD_ are 0.0008 (0.02 M) and 0.009 (0.04 M), and for Sox2 they are 0.0005 (0.02 M) and 0.002 (0.04 M). Oct1 was simulated with tethered domains, but POU_HD_ and POU_S_ were analyzed separately based on the center of mass (COM) of each domain. *B*, MSD profiles for SAP1 (*red*) and SRF (*blue*) at 0.02 M and 0.04 M, analyzed as described in panel A. Standard errors for SAP1 are 0.0003 (0.02 M) and 0.004 (0.04 M), and for SRF they are 0.0002 (0.02 M and 0.04 M). *Bottom panels*: Schematics illustrate representative TF diffusion behaviors projected onto the DNA. The trajectories capture sliding (tight, continuous contact), hopping (intermittent binding), and 3D motion (detachment and rebinding), providing visual insights into the distinct diffusion modes used by each TF during non-specific DNA search. TF, transcription factor.

MSD analysis of the transcription factors in the Sox2·Oct1·DNA system (Fig. 6*A*) reveals distinctly different diffusion behaviors along non-specific DNA for Sox2 compared with the POU_HD_ domain of Oct1, offering insight into how the mobility of each TF may contribute to the observed binding mechanism in which Sox2 consistently binds first and facilitates Oct1 recruitment. At 0.02 M salt, Sox2 exhibits a linear diffusion coefficient (D_1_) that is half that of POU_HD_, suggesting different nonspecific DNA-binding affinities. This slower diffusion means that Sox2 is less likely to miss or skip over its target cognate site during scanning, thus increasing the probability of it initiating binding. When the salt concentration is increased to 0.04 M (Fig. 6*A*, inset), POU_HD_ shows greater variability in trajectory slopes, and its linear diffusion decreases from 100% to 75%, while Sox2 maintains complete linear diffusion. The higher salt concentration leads to increased D_1_ values for both proteins. At 0.04 M, the value of D_1_ for Sox2 becomes only 1.7 times higher than that of POU_HD_, a smaller ratio than observed at 0.02 M. However, since POU_HD_ spends 25% of its time in 3D diffusion at 0.04 M, the overall difference in motion between Sox2 and POU_HD_ is actually more pronounced compared with their behavior at 0.02 M. Compared with Sox2, Oct1’s POU_S_ domain engages in weaker electrostatic interactions with DNA, with this reflected in its distinctly different scanning behavior. At 0.02 M, only 65% of its movement occurs through linear diffusion (data not shown), making D_1_ measurements unreliable. At 0.04 M, POU_S_ predominantly employs 3D diffusion (70%) to locate nonspecific DNA, further diminishing its use of linear diffusion mechanisms. These results indicate that POU_S_ has difficulty maintaining stable DNA interactions, highlighting the important function of Sox2 in recruiting POU_S_ to stabilize the formation of the ternary complex.

The bottom panel of Figure 6*A* demonstrates these contrasting scanning behaviors at a 0.02 M salt concentration. Sox2 primarily uses a sliding motion along the DNA backbone, consistently with its strong DNA-binding affinity. POU_HD_ relies mainly on hopping mechanisms, reflecting its weaker DNA interactions. POU_S_ displays both hopping and three-dimensional diffusion, where the 3D movement indicates the weakest DNA affinity among the three proteins, even under these low-salt conditions. These distinct DNA search modes reflect how each domain’s binding affinity influences its movement while searching for target sites on DNA. Further analysis of the TFs motions is shown in Figure S6.

For the SRF⋅SAP1⋅DNA system, we aimed to gain further insight into how a TF can function both as a recruiter and as a recruited protein, and how the DNA conformation determines which role each TF adopts. By examining the dynamics of each TF’s search for its cognate site and analyzing the diffusion behavior of SRF and SAP1 along nonspecific DNA, we sought to identify whether they share common characteristics in their search mechanisms.

At a 0.02 M salt concentration, both SRF and SAP1 clearly exhibit sliding motions along DNA (bottom panel, Figs. 6*B*, S7). Under these conditions, the values of D_1_ are 0.011 Å^2^ ps^-1^ for SAP1 and 0.010 Å^2^ ps^-1^ for SRF (Fig. 6*B*), indicating strong affinity for non-specific DNA and sufficiently slow diffusion to enable either TF to complex independently with the DNA. However, at a higher salt concentration of 0.04 M, despite both proteins maintaining the 100% linear diffusion behavior that enables them to complex independently with the DNA, their diffusion dynamics diverge. Under these conditions, SAP1 diffuses more rapidly, whereas the MSD of SRF shows minimal changes, suggesting that SRF retains a stronger affinity for DNA. This interpretation is further supported by the D_1_ values, with SRF retaining a low D_1_ of 0.011 Å^2^ ps^-1^, whereas SAP1 increases to 0.018 Å^2^ ps^-1^. This increase in SAP1’s linear diffusion coefficient indicates a weaker affinity for non-specific DNA, leading to more frequent hopping events compared with SRF. In contrast, SRF exhibits stronger electrostatic interactions, likely due to its highly charged DNA-binding motifs and adjacent tails. These interactions suggest a non-specific DNA sampling approach, implying that SRF might preferentially bind to bent DNA conformations as a distinguishing mechanism. In contrast, when the DNA conformation is linear, SRF may struggle to distinguish its specific binding site and therefore relies on SAP1 to compensate for the suboptimal DNA conformation.

### Protein–protein interactions

The two ternary systems we investigated, Sox2·Oct1·DNA and SRF·SAP1·DNA, are both characterized by direct protein–protein interactions between the 2 TFs, as observed in their crystal structures. Our initial hypothesis was that these interactions reflect functional cooperativity, where 1 TF assists the other in binding to its target site, thereby facilitating formation of the full ternary complex. However, our results revealed that the nature and functional importance of these interactions differ between the two systems.

In the Sox2·Oct1·DNA complex, the key interaction occurs between the DNA-binding domain of Sox2 and the POU_S_ domain of Oct1 (Fig. 1*A*). The POU_S_ domain exhibits very low affinity for DNA and has difficulty independently locating and stably binding its target site. Thus Sox2, which binds DNA more efficiently, plays a critical cooperative role by stabilizing POU_S_ at the correct position and enhancing ternary complex formation. In this system, the protein–protein interaction is essential to overcome the weak DNA-binding capability of one partner, highlighting a clear case of functional cooperativity.

In contrast, the SRF·SAP1·DNA system presents a different scenario. Here, the difference in DNA-binding affinity between the 2 TFs is less pronounced, reducing the need for cooperative assistance. Both SRF and SAP1 are capable of independently forming stable binary complexes with DNA. Structural analysis of the SRF·SAP1·DNA complex reveals three distinct protein–protein interfaces (Fig. S4): the B-box domain of SAP1 contacting SRF's MADS domain; interactions between their respective DNA-binding regions; and contacts involving their N-terminal tails. Among these, the B-box–MADS interface is thought to play a dominant role in stabilizing the interaction, with additional contributions from the DNA-binding motifs (52). However, in our CG-MD simulations, the B-box–MADS interface was poorly formed. This may be due to the high entropic cost associated with the disordered linker connecting SAP1’s domain to its B-box, which restricts stable interface formation. Given that both TFs already have high intrinsic affinity for DNA, we propose that this protein–protein interface may function primarily by reducing the dissociation rate of the ternary complex, rather than by promoting its association.

### Mechanism of ternary complex formation: comparison between computational and experimental results

#### Formation mechanism of the Sox2⋅Oct1⋅DNA ternary complex

**Figure 7:**
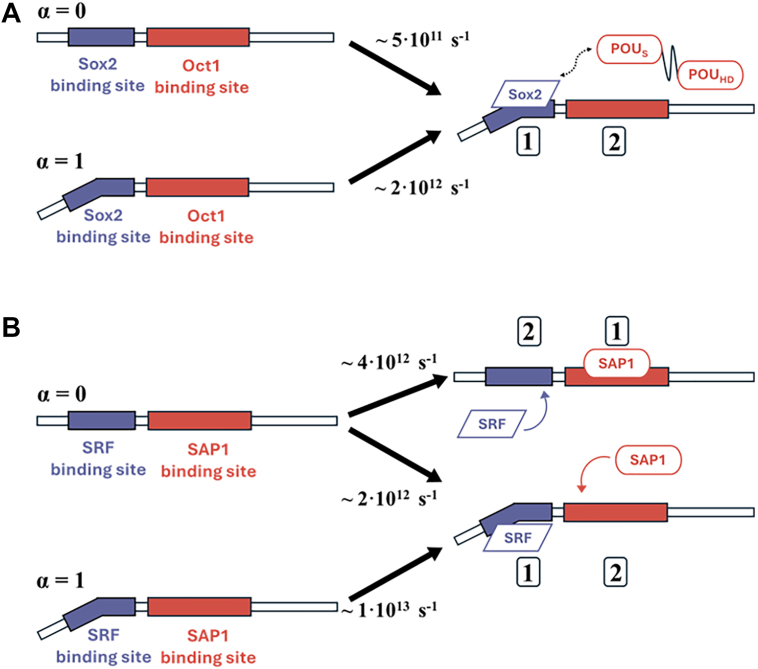
**Schematic representation of mechanisms of ternary complex formation influenced by DNA conformation**. The mechanisms for the Sox2·Oct1·DNA (*A*) and SRF·SAP1·DNA (*B*) systems are based on the coarse-grained simulations. The parameter α represents DNA conformation, with the extreme cases shown: α = 0, where DNA tends to adopt a straight B-DNA conformation, and α = 1, where DNA is highly bent. The rates above the *arrows* indicate the $k_{on}^{Ternary}$ association rate. The numbers one and two denote proteins that bind first and second, respectively. In the Sox2·Oct1·DNA system, the binding order is independent of DNA conformation. For all cases, Sox2 binds first, facilitating the binding of the POU_S_ domain of Oct1 (*dashed arrow*). In contrast, the SRF·SAP1·DNA system exhibits a DNA conformation-dependent mechanism. When the DNA adopts the B-DNA conformation (α = 0), two binding scenarios exist, with the faster mechanism favoring SAP1 binding first. However, when the DNA is bent (α = 1), the mechanistic preference is reversed, with SRF-first binding being significantly favored. TF, transcription factor.

Our kinetic analysis based on the CG-MD simulations revealed that the optimal pathway for ternary complex formation begins with Sox2 binding, followed by Oct1 (Fig. 7). This mechanism is supported by the binary association rates, with Sox2 showing faster DNA-binding kinetics than Oct1. Moreover, pre-bound Sox2 is shown to approximately double the association rate of Oct1 to DNA. These findings from the CG-MD simulations align with previous experimental kinetic studies, which demonstrated that Sox2 initiates Oct1⋅Sox2⋅DNA ternary complex formation (56, 57). Those experiments showed that binary Sox2·DNA complex formation occurs approximately 10 times faster than Oct1·DNA complex formation. Furthermore, once Sox2 is bound, it facilitates Oct1 binding to the adjacent site through protein–protein interactions, accelerating this process 13-fold (56). From the ternary and binary rate constants, the cooperativity between the two proteins can be estimated as *kT*ln(*k*^*ternary*^*/k*^*binary*^) which is about 1.5 kcal/mol.

The differences in binary association rates and the necessity for pre-bound Sox2 to facilitate Oct1 binding can be explained by the distinct DNA-binding characteristics of these proteins when interacting with nonspecific DNA sequences. Oct1 contains two DNA-binding domains: POU_S_ and POU_HD_. The POU_S_ domain demonstrates weak binding to nonspecific DNA sequences, as indicated by its low usage of sliding or hopping while scanning the DNA *via* linear diffusion, whereas the POU_HD_ domain demonstrates moderate affinity to nonspecific DNA, characterized by it also adopting a linear hopping motion (Fig. 6*A*, lower panel). This difference between the DNA affinities of the two domains has been observed both experimentally (58) and through simulations (59). In contrast, Sox2 exhibits a significantly higher affinity for nonspecific DNA and diffuses along the DNA following sliding dynamics. This difference highlights Oct1's reliance on interactions with Sox2 to strengthen its DNA binding and establish a stable ternary complex. A similar requirement for a secondary TF to form a stable ternary complex is observed in the Pax5·Ets-1·DNA system (60). Like Oct1, the Pax5 protein contains two DNA-binding subdomains with distinct binding properties: the CTD domain binds nonspecific DNA sequences, whereas the NTD domain interacts only weakly with nonspecific DNA sites. Similar to the Sox2·Oct1 system, protein–protein interactions between Ets-1 and the NTD of Pax5 enable the weaker DNA-binding domain to participate in forming a stable ternary complex, even at suboptimal binding sites.

The ability of Sox2 to bind specific DNA further supports the Sox2-first binding mechanism. Our kinetics results indicate that DNA bending significantly enhances the efficiency of Sox2·DNA binary complex formation. When DNA adopts its specific bent conformation, the association rate for Sox2 binding increases approximately threefold compared with when DNA remains in the B-DNA conformation (Fig. 2*B*). This acceleration aligns with previous studies emphasizing the crucial role of DNA bending in Sox2 recognition. It was demonstrated that Sox protein function relies on precise DNA bending to establish the structural framework necessary for transcriptional machinery operation (61). DNA structural flexibility is key to the functioning of Sox2, as demonstrated by MD simulations of sex determining region Y, another member of the Sox protein family. These simulations show that DNA sequences with greater intrinsic deformability toward the bound conformation exhibit higher binding affinity to the protein (27). This preferential binding of pre-bent DNA sequences enables Sox2 to act as a pioneer factor, allowing it to bind silent, nucleosome-enriched DNA and facilitate the recruitment of additional TFs (62).

#### Formation mechanism of the SRF⋅SAP1⋅DNA ternary complex

The optimal mechanism for forming the SRF·SAP1·DNA ternary complex, as suggested by the kinetic analysis of the CG-MD simulations, depends on the inherent dynamics that determine the DNA conformation (Fig. 7*B*). On linear DNA, the faster pathway involves SAP1 binding first, followed by the recruitment of SRF. However, as the DNA sequence becomes more deformable and more likely to adopt the required bent conformation, the difference between the rates of these two binding order mechanisms diminishes. At highly bent DNA conformations, SRF transitions from a passive binder to an active recruiter, facilitating SAP1 binding. This variation in binding mechanisms across different DNA sequences has also been observed in experimental studies (63). As mentioned in the introduction, SRF recruits a TCF, such as SAP1, to its respective DNA sites to fully activate the SRE of the proto-oncogene c-fos. However, when mutations occur at the SRF binding site and a specific binding site for the TCF is present, the roles of the recruiter and recruited are reversed, with the TCF now facilitating SRF binding. This sequence-dependent shift in mechanism can be attributed to SRF’s requirement for a bent DNA conformation to facilitate binding. The native c-fos SRE sequence naturally adopts a stable hairpin structure, providing the necessary bent conformation that enhances SRF recognition (64). In contrast, mutations that disrupt this pre-bent structure have been shown to reduce SRF binding significantly (65), thereby increasing its reliance on cooperative interactions with another transcription factor, such as TCF. These findings further support our results that, in highly bent DNA conformations, SRF acts as the recruiter, whereas in less bent conformations, a pre-bound SAP1 facilitates SRF binding, making ternary complex formation more efficient.

## Discussion

In this study, we investigated the molecular mechanisms of ternary TF·TF·DNA complexes using CG-MD simulations. Our objective was to address fundamental questions regarding ternary complex assembly, such as the molecular determinants that dictate the kinetics of complex formation and particularly the role played by DNA conformation in these processes. We examined two ternary complexes with seemingly similar structural characteristics, each containing 2 TFs bound to DNA. In both cases, 1 TF binds to a bent DNA region, while the other interacts with a straighter, more B-DNA-like segment, forming a protein–protein interface. Our study shows that the mechanisms and kinetics of the formations of these two ternary complexes share similar features, yet they also possess distinct driving forces that underly their molecular mechanisms.

In both systems, the order in which the 2 TFs bind to DNA is primarily dictated by their interaction with nonspecific DNA, which influences the dynamics of their search for their specific DNA site. The TF that has a higher diffusion coefficient is less likely to initiate the formation of the ternary complex. Instead, the TF that binds first is the one with a higher affinity for both nonspecific and specific DNA, regardless of whether the DNA undergoes a conformational change. This finding contradicts our initial hypothesis, which suggested that DNA bending would determine the mechanism of ternary complex formation. We initially expected that TF binding would be less favorable when the TF requires a bent binding site due to the higher energetic cost, and would therefore require assistance from a pre-bound TF to overcome this energetic barrier. Consequently, we expected this would regulate ternary complex formation. However, our results indicate that the rate of ternary complex formation depends on multiple factors, including the properties of the TFs, the DNA, and the nature of their nonspecific and specific interactions. As a result, conformational changes do not always dictate the kinetics of ternary complex formation between TFs and DNA.

In the Sox2·Oct1·DNA system, the markedly different affinities of the 2 TFs for non-specific DNA create a cooperative mechanism in which Oct1 binding to DNA is supported by the Sox2 protein, consistently with the experimental kinetic results (56). Sox2, with its preference for linear diffusion and slower movement along DNA, typically finds, recognizes, and binds first despite the fact that it binds to a bent DNA sequence and thus may need to overcome an energetic barrier to achieve the deformed conformation. Sox2 then facilitates Oct1 binding through two key mechanisms: by reducing the diffusion rate of POU_S_ along non-specific DNA, thereby increasing the likelihood of binding site recognition, and by forming stabilizing protein–protein interactions once POU_S_ reaches its target site. This cooperative recruitment is crucial for overcoming the inherent instability of POU_S_–DNA interactions.

The SRF·SAP1·DNA system demonstrates a distinct mechanism where both TFs possess a relatively strong intrinsic affinity for DNA. This characteristic enables both SRF and SAP1 to bind autonomously to their DNA binding site, without requiring assistance from another protein, making the binding order more dependent on site-specificity and therefore more influenced by DNA conformation. When the DNA is more bent, SRF binds to its binding site significantly faster than SAP1, leading to sequential assembly whereby SRF binds first followed by SAP1. However, the competition between the SRF and SAP1 TFs intensifies when the DNA approaches a B-form conformation, which reduces the specificity of SRF's cognate binding site and thereby disrupts SRF's recognition process. Under these conditions, the system can adopt an alternative binding order in which SAP1 binds first. This role reversal in recruitment has also been reported experimentally, where mutations in SRF’s binding site prevent its optimal binding, allowing SAP1 to recruit SRF instead (63). This illustrates how the interplay between DNA conformation and protein–DNA affinity can influence assembly pathways. This also highlights the combinatorial control characteristic of higher organisms, where variations in DNA structure and binding affinities enable dynamic regulation of transcription factor binding, allowing for context-dependent gene expression.

This study highlights the interplay between the molecular properties of TFs, the DNA sequence and its deformability, and protein–protein interactions, all of which shape the kinetics and molecular mechanisms of ternary complex formation between TFs and DNA. In some systems, the combined influence of these factors leads to a robust and well-defined mechanism of complex formation. In others, modifications to the molecular components (*e*.*g*., mutations) or changes in environmental conditions (*e*.*g*., salt concentration) can alter these interactions, thereby modulating the mechanism of ternary complex formation. This complexity in assembly mechanisms likely contributes to the versatility and specificity of transcriptional regulation in biological systems.

Given that our study focuses on only two systems, other systems may follow alternative mechanisms influenced by various factors, including differences in TF conformational dynamics, DNA flexibility, cooperative interactions, and environmental conditions. Moreover, cooperativity may arise not only from direct protein–protein interactions but also through allosteric effects mediated by the DNA, whereby the binding of one protein affects the DNA-binding site of another (66, 67, 68). Further research is needed to explore additional mechanisms governing ternary complex formation and the formation of higher-order protein–DNA complexes to achieve a better understanding of how combinatorial control contributes to transcriptional regulation across different biological contexts.

## Experimental procedure

### Molecular systems of the studied ternary complexes

To study the kinetics and mechanisms of ternary complex formation, as well as the impact of DNA conformation on these processes, we studied two ternary complex systems, each of which involves two proteins binding to a DNA segment: Sox2⋅Oct1⋅DNA (PDB ID 1O4X) (29) and SRF·SAP1·DNA (PDB ID 1HBX) (52) (see Fig. 1). For each system studied, the TF structures were obtained from the available solved ternary complex. Missing linker residues or disordered tails were reconstructed using the Modeller program (69) and treated as flexible regions during simulations (*via* reduction of angle and dihedral parameters). The DNA coordinates were extracted from the solved structure and subsequently extended to a total length of 100 random sequence base pairs, with the additional segments modeled as canonical B-DNA using the x3DNA webserver (70) (see Fig. 1). The extended sequence was random, since the sequence information is lost in modeling of nonspecific DNA that are modeled uniformly solely by electrostatic interactions. Through introduction of uniform parameters across every nucleotide.

To achieve a molecular understanding of the mechanism of complex formation, each of the studied ternary complexes was investigated under four scenarios. Two scenarios involved the formation of binary complexes between each TF and DNA, whereas the other two scenarios involved the formation of a ternary complex between both TFs and DNA, with modelling focused on the free diffusion and binding of the second TF to a DNA strand to which the first TF was already prebound. Ternary complex formation was explored twice, switching between identities of the freely diffusing and prebound TFs (see Fig. 2, right panel).

### Protein modeling

In the CG model employed in this study, each protein residue is represented by a single bead positioned at the center of the Cα atom. The force field used in these simulations implements a native-topology-based potential for the proteins, where the experimentally determined structure serves as the global energy minimum during the simulations. This approach has been applied previously in simulations of other protein–DNA interactions (55, 71). The native protein structure, serving as the global minimum, is described using a specific set of parameters modeled by different types of potentials. Bonds and angles are represented by harmonic potentials, while dihedral angles are modeled using periodic potentials. Native contacts are modeled using a Lennard-Jones potential. They are classified into intra-native contacts, which exist within each protein and define its internal structure, and inter-native contacts, which occur between biomolecules and govern interactions at the interface. Both types of contacts are identified using the contacts of structural units algorithm, based on the PDB file that defines the native structure and used in analysis to achieve specific binding of the TFs to DNA. Additionally, repulsive interactions are introduced for protein bead pairs that do not participate in native contacts, thus preventing non-native interactions or bead overlap. The final potential is an electrostatic interaction between charged groups, modeled using the Debye-Hückel potential. The total potential energy for a given protein conformation during the MD simulation trajectory is calculated as the sum of all these contributions.

$$V\left(\Gamma ,\Gamma 0\right)=\sum_{\text{bonds}}k_{\text{ij}}^{b}{\left(r_{\text{ij}}-r_{\text{ij}}^{0}\right)}^{2}+\sum_{\text{angles}}k_{\text{ijk}}^{a}{\left(\theta_{\text{ijk}\;}-\theta_{\text{ijk}}^{0}\right)}^{2}+\sum_{\text{dihedrals}}k_{\text{ijkl}}^{d}\left[\left(1-\cos\left(\phi_{\text{ijkl}}-\phi_{\text{ijkl}}^{0}\right)\right)+\frac{1}{2}\left(1-\cos\left(3\left(\phi_{\text{ijkl}}-\phi_{\text{ijkl}}^{0}\right)\right)\right)\right]+\sum_{\begin{array}{c} \text{contacts}\\ i\neq j \end{array}}\in_{\text{ij}}^{C}\left[5{\left(\frac{\sigma_{\text{ij}}}{r_{\text{ij}}}\right)}^{12}-6{\left(\frac{\sigma_{\text{ij}}}{r_{\text{ij}}}\right)}^{6}\right]+\sum_{\begin{array}{c} \text{repulsions}\\ i\;\\ \text{lt};\;j-4 \end{array}}\in_{\text{ij}}^{\text{NC}}{\left(\frac{\sigma_{\text{ij}}}{r_{\text{ij}}}\right)}^{12}+\sum_{\text{ij}}K_{\text{Coulomb}}B\left(\kappa \right)\;\left[q_{i}q_{j}\frac{e^{-\kappa r_{\text{ij}}}}{\epsilon_{bulk}r_{\text{ij}}}\right]$$

The potential energy of a given conformation V(Γ, Γ_0_) is determined by the parameters of the specific conformation (Γ) and the native conformation (Γ_0_). The force constants for bonds ($k_{\text{ij}}^{b}$), angles ($k_{\text{ijk}}^{a}$), dihedrals ($k_{\text{ijkl}}^{d}$), intra-native contacts ($\epsilon_{\text{ij}}^{C}$), and repulsions ($\epsilon_{\text{ij}}^{\text{NC}}$) are set to 100 kcal mol^-1^ Å^-2^, 20 kcal mol^-1^, 1 kcal mol^-1^, 1 kcal mol^-1^, and 1 kcal mol^-1^, respectively, in order to maintain the experimental protein structure. The linker and tails residues were identified as intrinsically disordered regions (IDRs), and their force constant values were reduced to 0 kcal mol^-1^. Additionally, intra-native contacts for these regions are removed.

The inter-native contact constant ($\varepsilon_{ij}^{C}$) is system-specific and varies among proteins and between different protein–DNA complexes to optimize sampling of binding and dissociation events. For each system, $\varepsilon_{ij}^{C}$ was adjusted to yield at least two dissociation events, ensuring sufficient sampling for reliable estimation of kinetic parameters. The distinct $\varepsilon_{ij}^{C}$ values reflect differences in complex stability. This procedure enables meaningful comparison of binary and ternary binding within a given system but limits direct comparison across systems. In the SRF·SAP1·DNA system $\epsilon_{\text{ij}}^{C}$ is set to 0.2 kcal mol^-1^ when the proteins are free to diffuse along the DNA and is set to 1 kcal mol^-1^ when the proteins are fixed at their binding sites. In the Sox2⋅Oct1⋅DNA system, $\epsilon_{\text{ij}}^{C}$ is 0.75 kcal mol^-1^ for free diffusion along DNA, and set to 1 kcal mol^-1^ for Sox2 and 7 kcal mol^-1^ for Oct1 when fixed at their binding sites.

The total potential energy for a given conformation includes terms for bonds (r_ij_, the distance between sequential beads i-j), angles (θ_ij_, the angle in radians between sequentially bonded beads i-j-k), and dihedral angles (ϕ_ijkl_, the angle in radians between four sequentially bonded beads i-j-k-l). Parameters derived from the native structure are denoted with a superscript 0 (*e*.*g*., $r_{\text{ij}}^{0}$). The distance σ_ij_ between non-sequential interacting beads is also based on the native structure.

The electrostatic interaction term in the energy function is modeled using a Debye–Hückel potential, which is determined by the distance between charged beads (r_ij_) and includes a Coulomb constant (K_Coulomb_ = 332 kcal mol^-1^e^-2^). The total charges of residues (q_i/j_) are assigned as follows: −1 for negatively charged beads (Asp, Glu, and DNA phosphate groups), +1 for positively charged beads (Arg and Lys), and 0 for neutral beads. The dielectric constant (ϵ_bulk_) is set to 80, κ is the screening factor, and B(κ) is a salt-dependent coefficient.

#### DNA modeling

The DNA molecules were simulated using the recently developed deformable DNA model (27). This model is adapted for CG-MD simulations and enables control of gradual DNA transitions between two conformations, by controlling the distance between phosphate beads on the DNA. Notably, the model does not account for DNA sequence specificity and instead applies uniform parameters to all nucleotides. Sequence-dependent behavior is represented indirectly through differences in deformability, which capture the varying propensities of DNA segments to adopt or transition between conformational states. Accordingly, references to “sequences” in this study should be understood as abstractions of distinct deformability profiles rather than variations in the primary nucleotide sequence. To achieve transitions between two DNA conformations, a dual-basin Gaussian potential (dbG) was applied. This potential allows the DNA’s geometric parameters to adopt two extracted values corresponding to two conformations (*e*.*g*., bent and linear DNA). The dbG values were assigned to phosphate–phosphate pairs, from the original bent DNA, that exhibit significant distance differences between the linear and bent states. In the absence of external factors, such as proteins or ions, the probability of sampling the DNA in any particular conformation along the linear–bent transition depends on the depths of the corresponding energy basins, which are represented by parameters ϵ_linear_ and ϵ_bent_, whose relative magnitudes determine DNA sequence deformability. In this study, ϵ_bent_ was kept at a constant value of 6, while ϵ_linear_ was varied. Additional details of the dual-basin Gaussian can be found in the supporting information.

The extent to which the DNA sequence adopts a bent conformation is described by the α parameter, which is quantified as the fraction of phosphate–phosphate pairs in the dbG set whose distances match those defining the bent structural conformation. Higher α value indicate a greater bent-state population, with nearly perfect DNA bending achieved at α ≈ 1. To ensure a full range from 0 to 1, a slightly different set of ϵ_linear_ values was required for each system. For each system, we used six values of ϵ_linear_. The value of α that is associated with each ϵ_linear_ was obtained as the mean from five independent 5-μs DNA simulations. The relationship between the ϵ_linear_ and α values for the two studied systems is provided in Figure S1. Each kinetic scenario was analyzed through 50 simulations using six values of α, ranging from 0 to 1. The temperature was maintained at 0.45, and the salt concentration was set to 0.04 M. The salt concentration was selected to be low to avoid dissociation of the TFs from the DNA which would make the sampling more challenging but is not expected to affect the cooperativity between the two proteins that form the ternary complex with DNA. Each simulation ran for 10^8^ steps (5 μs), with trajectory data collected every 1000 steps (corresponding to 50 ps).

#### Analysis of the kinetics and thermodynamics of ternary DNA complexes

Kinetic parameters for each system were calculated using Q_Binding_, representing the fraction of contacts formed between two biomolecules. For DNA–protein kinetics, Q_Binding_ specifically quantifies the fraction of native contacts determined by contacts of structural units algorithm, formed between the protein and the DNA. Based on their Q_Binding_ values, protein–DNA interaction states were categorized as: the search state (S), in which no specific binding occurs; the intermediate state (I), representing partial binding; and the recognition state (R), indicating full binding at the target site. Contact determination relied on measuring distances between specific bead pairs (i and j) in the molecular structure, with a contact being registered when the distance remained within 150% of the original distance as per the native structure. The Q_Binding_ values were calculated at each trajectory step and subsequently smoothed using a 500-step window to identify clear association and dissociation events. System-specific thresholds were established to differentiate between binding states. For the Sox2⋅Oct1⋅DNA system, the states were defined as: S state (Q_Binding_ = 0), R state (Q_Binding_ > 0.65), and I state (0 < Q_Binding_ < 0.65). In the case of Oct1, kinetic analysis focused specifically on interactions between the POU_S_ domain of Oct1 and DNA. The SRF⋅SAP1⋅DNA system utilized different binding state thresholds, with the R state defined as Q_Binding_ > 0.3 and the I state as 0 < Q_Binding_ < 0.3.

In each case, the protein was positioned approximately one DNA helical turn (∼30 Å) away from their target sites, in the direction opposite to the second protein’s binding site. Kinetic parameters were derived from the trajectory analysis: The association rate constant (k_on_) was measured as the inverse of the mean first passage time from the S to R state that followed the first dissociation rate (first binding event was ignored as it is depends on protein positioning relative to the target site), whereas the dissociation rate constant (k_off_) was calculated as the mean first passage time from the R to S state. The thermodynamic dissociation constant (K_D_) was computed as the ratio $\frac{k_{\text{off}}}{k_{\text{on}}}$ (see Fig. S2). The association rate constant for ternary complex formation DNA⋅X⋅Y for a reaction that protein X binds before protein Y can be expressed as $k_{\text{on}}^{\text{Ternary}}∼k_{\text{on}}^{\text{proteinX}}{\cdot k}_{\text{on}}^{\text{proteinY}\cdot \text{proteinX}}/\left(k_{\text{off}}^{\text{proteinX}}+k_{\text{on}}^{\text{proteinY}\cdot \text{proteinX}}\left[Y\right]\right)$, where $k_{\text{on}}^{\text{proteinX}}$ represents the binary association rate of protein X with DNA, and $k_{\text{on}}^{\text{proteinY}\cdot \text{proteinX}}$ represents the ternary association rate of protein Y with DNA when protein X is pre-bound to its DNA binding site. We estimate the rate constant of the ternary reaction in two scenarios. In the first scenario we assume that the two reactions of binding of proteins X and Y are independent (*i*.*e*., weak cooperativity) and therefore $k_{\text{on}}^{\text{Ternary}}$ is given by $k_{\text{on}}^{\text{proteinX}}{\cdot k}_{\text{on}}^{\text{proteinY}\cdot \text{proteinX}}$. In the second scenario, we assume that the complex between DNA and protein X is a preequilibrium before protein Y binds. In this case, $k_{\text{off}}^{\text{proteinX}}>k_{\text{on}}^{\text{proteinY}\cdot \text{proteinX}}\left[Y\right]$, which gives $k_{\text{on}}^{\text{Ternary}}∼k_{\text{on}}^{\text{proteinX}}{\cdot k}_{\text{on}}^{\text{proteinY}\cdot \text{proteinX}}/k_{\text{off}}^{\text{proteinX}}$.

The skipping value quantifies the mean number of unproductive binding attempts that occur before full binding (R state) is achieved. Unproductive binding (*i*.*e*., skipping) is defined as transitions from the S to I state that revert to the S state without progressing to full binding in the R state. All uncertainties were quantified using the SEM.

#### Characterization of protein search along DNA

The diffusion coefficients for the linear diffusion of proteins along DNA were determined from the MSD of the protein's center of mass (COM), calculated using the equation: $\sum_{i=t_{0}}^{t-\tau }\frac{{\left(r_{i+\tau }-r_{i}\right)}^{2}}{t-\tau }=2\text{dD}\tau$ (72), where r is the position of the protein's COM, t is the number of time steps measured, and τ is the measurement window ranging from t_0_ to t. The slope of the MSD is 2dD, where d is the dimensionality of diffusion and D is the diffusion coefficient, which was calculated between timeframes 1 and 200 (where each time frame consists of 1000 steps, corresponding to 50 ps long (73)).

We calculated the linear diffusion coefficient (D_1_) specifically by setting the diffusion dimensionality to d = 1, with the direction defined along the DNA axis (x-axis). The calculation was restricted to conditions where at least 70% of the diffusion occurred linearly, defined as the TF being within 32 Å of the DNA. Distances beyond this threshold were considered 3D diffusion and were excluded. Proximity to the DNA was quantified as the distance between the protein COM and the COM of the closest bead of DNA base. D_1_ was computed only for the time intervals corresponding to linear diffusion events occurring between time frames 100 to 200.

## Data availability

The Python code of custom functions used for data processing and analysis, including kinetic calculations and MSD analysis (functions.py) and the in-house MD simulation code used to generate all datasets analyzed in the paper (MD_dbGPotential-main.zip) can be found at https://doi.org/10.5281/zenodo.15838764. The code of dual-basin DNA can be found in https://github.com/elRogou/MD_dbGPotential.

## Supporting information

This article contains supporting information (27,74).

## Conflict of interests

The authors declare that they have no conflicts of interest with the contents of this article.
