# Supplementary material for: Ternary complexes in protein–DNA interactions: Kinetics and mechanisms
Source: J Biol Chem. 2025 Dec 8;302(1):111024. doi: 10.1016/j.jbc.2025.111024 (PMC12805347; doi:10.1016/j.jbc.2025.111024)
Supplement: Supplementary Material 1 [file mmc1.pdf]

Ternary Complexes in Protein–DNA Interactions: Kinetics and Mechanisms

Adina Hefetz, Elena Rogoulenko and Yaakov Levy

Supporting information

DNA modeling

The dual-basin Gaussian assigned to each phosphate-phosphate pair is defined as follows(1):

$$U_G = \epsilon\left[\left(1+\left(\frac{\sigma_{NC}}{r_{ij}}\right)^{12}\right)\left(1+G\left(r_{ij},r_1^{ij}\right)\right)\left(1+G\left(r_{ij},r_2^{ij}\right)\right)-1\right]$$

where, $G\left(r_{ij},r_{n=1,2}^{ij}\right) = -\epsilon_n \exp\left[-\frac{\left(r_{ij}-r_n^{ij}\right)^2}{2\sigma_n^2}\right]$ and r^{ij} denotes the distance between the identified pair of phosphate beads, while r_n^{ij} represents the distance between pairs of beads in a specific state. ϵ is a global scaling factor and was set to be 1 in our simulations. Here, n = 1 refers to the B-DNA structure and n = 2 denotes the original bent structure state. The parameter ϵ_n indicates the depth of the energy basin, which modulates the interaction strength. The excluded volume value, represented by σ_{NC}, was set at 0.4 nm. The width of each well was adjusted to $\sigma_n = r_1^{ij} - r_2^{ij}$. The model also allows additional conformations that constitute various combinations of the parameters of the two distinct conformations. In this study, the potential were applied on phosphate-phosphate pairs to obtain a relatively similar number of identified pairs across systems. The phosphate-phosphate pairs that differ in their distance over 20% in B-DNA structure and bound complex were represented by the dual-basin Gaussian potential. This resulted in 21 pairs for the Sox2·Oct1·DNA system and 27 pairs for the SRF·SAP1·DNA system.

The ability of DNA sequence to transition between two conformations is described by the α parameter, which quantified using the ratio between the energy wells that represent each conformation (detailed description of the model in https://doi.org/10.1093/nar/gkae333) . Higher α values indicate greater population of the bent state, with nearly perfect DNA bending achieved at $\alpha \approx 1$. To ensure a full range from 0 to 1, a slightly different set of ϵ_{linear} values was required for each system. For each system, we used six values of ϵ_{linear}. The value of α that is associated with each ϵ_{linear} was obtained as the mean from five independent 5 μs DNA simulations. The relationship between ϵ_{linear} and α

values for the two studied systems is provided in Figure S1. Each kinetic scenario was analyzed through 50 simulations using six values of α, ranging from 0 to 1. The temperature was maintained at 0.45, and the salt concentration was set to 0.04M. Each simulation ran for 10^8 steps (5 μs), with trajectory data collected every 1000 steps.

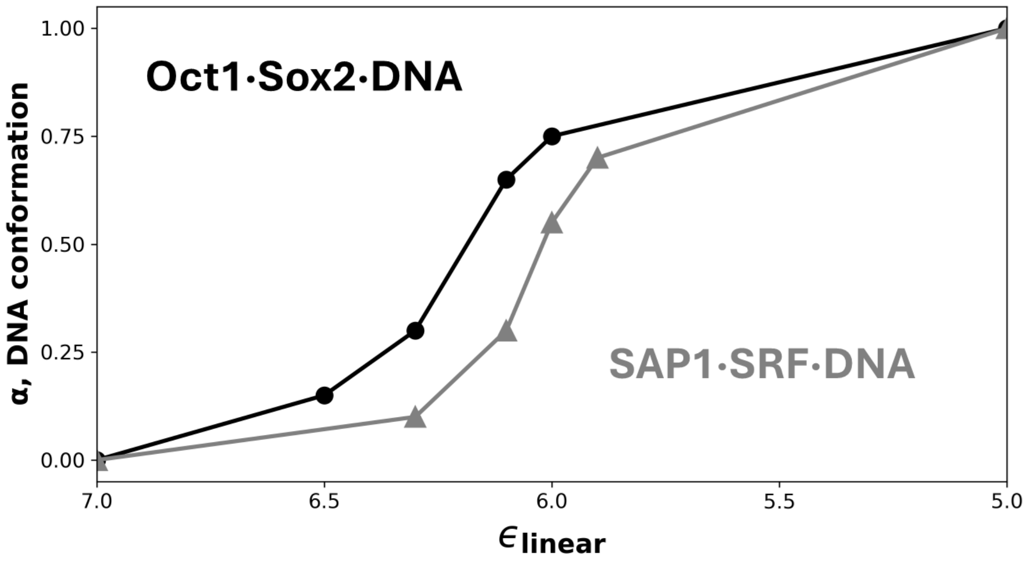

Figure S1. DNA conformations as a function of ϵ_{linear}. The α values represent the simulated DNA conformations and their corresponding ϵ_{linear} conditions. The α values range from 0 to 1, where α = 0 corresponds to a linear B-DNA conformation, and α = 1 represents a bent conformation derived from the crystal structure, specific to each system (Sox2·Oct1·DNA in black and SRF·SAP1·DNA in gray). Each α value was obtained as the mean fraction of phosphate–phosphate pairs in the dbG set whose distances match those defining the bent structural conformation, based on five independent 5 μs simulations performed under a specific ϵ_{linear} condition.

Thermodynamic equilibrium dissociation constant calculation

To quantify the differences between Sox2 and Oct1 TFs that may affect the mechanism of their ternary complex with DNA, we estimated the thermodynamic equilibrium dissociation constant (K_D). Here, we used K_D differences to estimate their binding affinities when each TF forms a binary complex with DNA. The K_D was estimated for the binding of TF with its specific DNA site with a being scanned between 0 and 1.

Our thermodynamic analysis shows that Oct1 (measured for the POU_S domain) exhibits a higher K_D than Sox2 when forming binary complexes with DNA, regardless of DNA conformation (Fig. S2). Initially, as DNA deviates from a linear form, the ratio rises sharply due to Sox2's increased affinity for its natural bent binding site. A gradual decline follows, driven by a slight increase in Oct1 affinity as DNA becomes more flexible, allowing better access to the major groove.

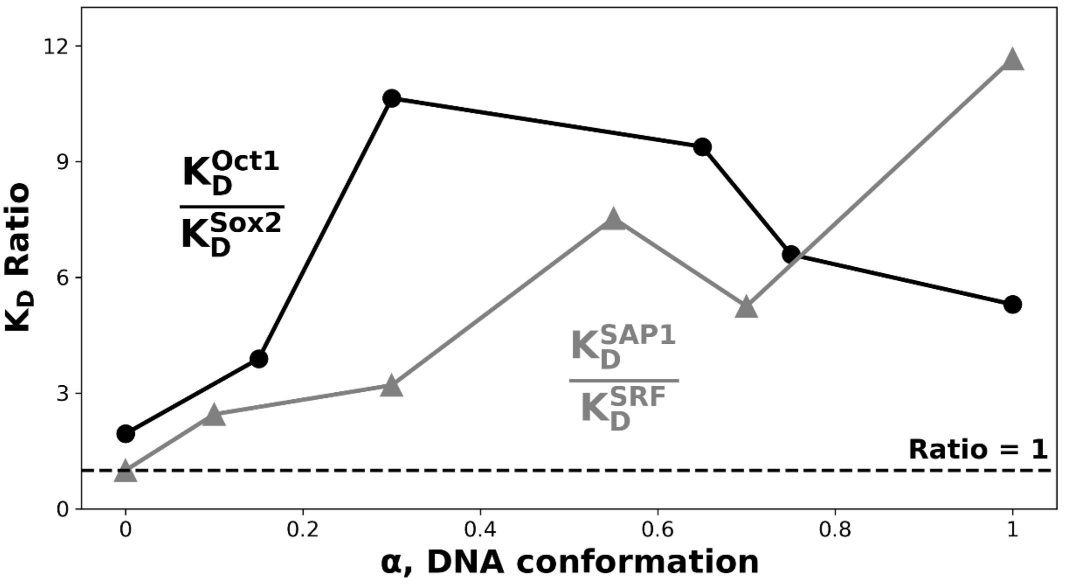

Figure S2. Relative DNA binding affinities across DNA conformations. Binary equilibrium dissociation constant for two transcription factor pairs: Oct1/Sox2 from the Sox2·Oct1·DNA system (black) and SAP1/SRF from the SRF·SAP1·DNA system (gray). Ratios above 1 indicate stronger binding affinity of the second protein (denominator) relative to the first protein (numerator), while ratios below 1 indicate stronger binding of the first protein. The dashed horizontal line at ratio = 1 represents equal binding affinities between protein pairs.

In the SRF·SAP1·DNA system, we also analyzed differences in K_D to determine whether variations in protein affinity for different DNA conformations could account for the observed trends and for the differing binary-complex formation rates of SRF and SAP1. The K_D ratio between SRF and SAP1, as estimated from CG-MD simulations, reveals notable differences in protein-DNA interactions across various DNA conformations (Fig. S2). For highly linear DNA, SRF and SAP1 exhibit similar binding affinities to DNA, with a ratio of approximately 1. However, as DNA becomes more bent, their affinities diverge significantly, leading to a sharp increase with SRF's affinity becoming 11 times stronger than SAP1's at highly bent DNA. This difference arises because SRF's affinity increases with DNA bending, whereas SAP1's affinity decreases, despite its original binding site not exhibiting a bent conformation.

The increased affinity of SRF for a bent binding site can be attributed to its electropositive density within its DNA-binding motifs as illustrated in the map electrostatic in Figure S3. A curved DNA structure carries higher electronegative density, which enhances SRF's retention at the site, reducing its dissociation rate (k_{off}). Additionally, the highly negatively charged bent DNA enhances SRF's recruitment to its cognate site by keeping it proximal to the binding site, thereby reducing the rebinding time and allowing it to bind more quickly and efficiently. Together, these factors lead to a lower K_D for SRF as the DNA adopts a bent conformation, ultimately influencing its association

kinetics.

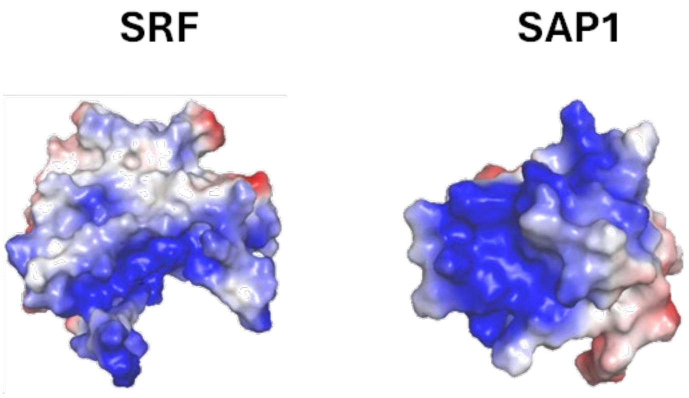

Figure S3. Electrostatic surface potential maps of SRF (left) and SAP1 (right). The color gradient represents electrostatic potential, with blue indicating positive potential and red indicating negative potential. In both proteins, the regions with high positive potential density correspond to the DNA-binding motifs.

Protein-protein interfaces in the SRF·SAP1·DNA complex

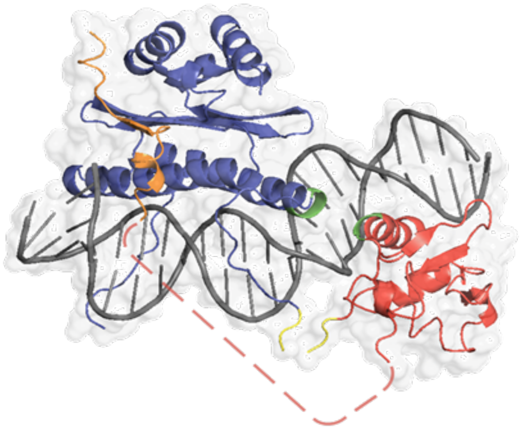

Figure S4. Structural representation of SAP1–SRF interactions. Structural representation of the full SAP1·SRF·DNA complex, highlighting the three distinct protein–protein interaction regions: DNA-binding motifs (green), B-box (orange), and N-terminal tails (yellow). The red dashed lines represent the disordered linker connecting SAP1's B-box to its core domain, which consists of 43 residues. This linker was not resolved in the experimental structures and was reconstructed using the Modeller program.

Characterization of DNA Search of the proteins prior to binding to specific DNA - coupling between rotation and translation

An alternative way to illustrate protein motion along DNA is by estimating the coupling between rotational and translational movements during linear diffusion. To do this, we tracked the position of the protein's center of mass (COM) along the DNA axis (X) and calculated the rotation angle (θ) between the protein and the DNA. The angle θ was computed in radians using the formula $\theta = \tan^{-1}(\frac{y}{z})$, where y and z are the coordinates of the protein's COM perpendicular to the DNA, which was aligned along the X-axis. A linear relationship, with slope approximately -0.18 $\frac{rad}{Å}$

between X and θ indicates a coupling between rotation and translation during protein diffusion along DNA, namely a sliding motion(2).

The ternary rate constant was estimated by assuming that the complex between DNA and protein X is in a preequilibrium before protein Y binds. In this case, $k_{off}^{proteinX} > k_{on}^{proteinY \cdot proteinX}[Y]$, which gives $k_{on}^{Ternary} \sim k_{on}^{proteinX} \cdot k_{on}^{proteinY \cdot proteinX} / k_{off}^{proteinX}$. This is different from the ternart rate constants shown in figure 4 that assumes $k_{on}^{Ternary} \sim k_{on}^{proteinX} \cdot k_{on}^{proteinY \cdot proteinX}$. The hierarchy of the ternary rate constants for the two systems as shown in Figure S5 is similar to that shown in Figure 4. This is particularly clear for the ternary complex between Oct1 and Sox2 to DNA, illustrating high preference for Sox2 to bind DNA before Oct1. For the binding between Sap1 and SRF to DNA, Figure S5 shows that for bent DNA the prefeered scenario is for SRF to bind before Sap1. For less bent DNA, the two pathways of ternary complex formations are more comparable.

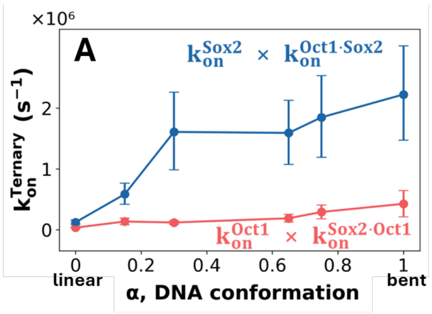

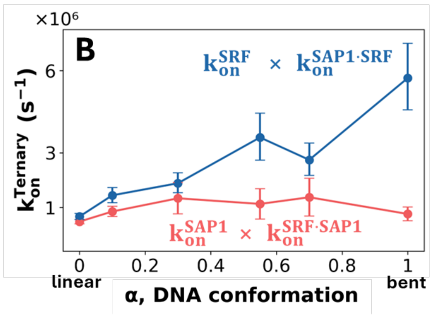

Figure S5. Overall rate of ternary complex formation for distinct binding mechanisms as a function of DNA conformation (α). The plot is similar to that shown in Figure 4 but the ternary rate constant is calculated following $k_{on}^{Ternary} \sim k_{on}^{proteinX} \cdot k_{on}^{proteinY \cdot proteinX}$.

Figure S6 presents the trajectories of Sox2 and of the tethered POU_S and POU_{HD} domains of Oct1 along nonspecific DNA in the [X,θ] space under 0.02M. Sox2 exhibits a clear sliding mechanism, characterized by a strong linear relationship between X and θ. Points aligning with the slope lines correspond to sliding, while points between the slopes indicate hopping. In contrast, the Oct1 domains display distinct diffusion behaviors. The POU_S domain, which interacts with Sox2 in the ternary complex (see Figure 1), exhibits a high percentage of 3D motion, with only 65% linear diffusion. Meanwhile, the POU_{HD} domain demonstrates approximately 100% linear diffusion (including both sliding and hopping motions) under the same salt concentration (0.02M). These differences emphasize potential variations in DNA interaction mechanisms between the two Oct1 domains.

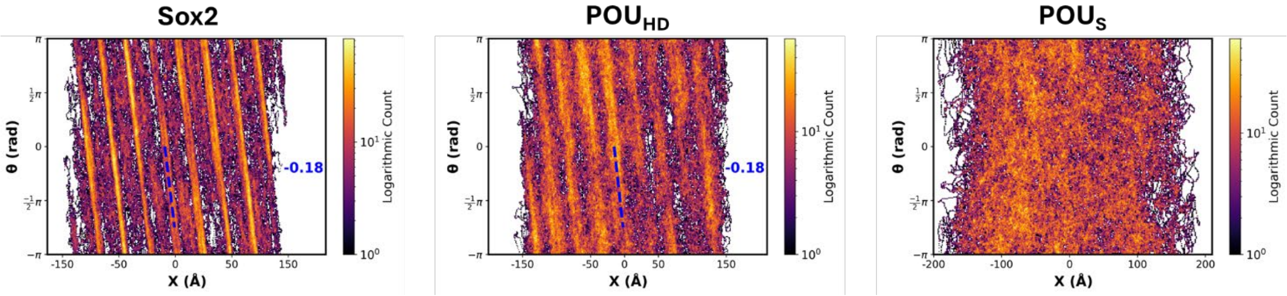

Figure S6. Heatmaps of protein diffusion trajectories in the [X,θ] space for Sox2, POU_{HD}, and POU_S. The motion mechanism is analyzed by projecting five sampled protein trajectories onto the [X,θ] space at a salt concentration of 0.02M, where X represents the center of mass (COM) position of the diffusing protein along the DNA axis, and θ denotes the protein's rotational angle. Sliding motion is characterized by a coupling between rotation and translation, revealed by a linear relationship between θ and X with an approximate slope of $-0.18\frac{rad}{Å}$ (dashed blue line). Scattered points deviating from the slope lines indicate diffusion via hopping or alternatively, engagement in 3D diffusion. Oct1 was simulated with both of its domains tethered together, but each domain was analyzed separately using its own COM.

Figure S7 illustrates the [X,θ] space for SAP1 and SRF proteins under 0.02M, highlighting their linear diffusion behavior through a clear linear relationship between X and θ. While both proteins exhibit sliding motion, SAP1 displays more scattered trajectories that deviate from the lines that correspond to coupled rotation-translation diffusion. This suggests that SAP1 diffuses faster as it involves more hopping than the SRF protein.

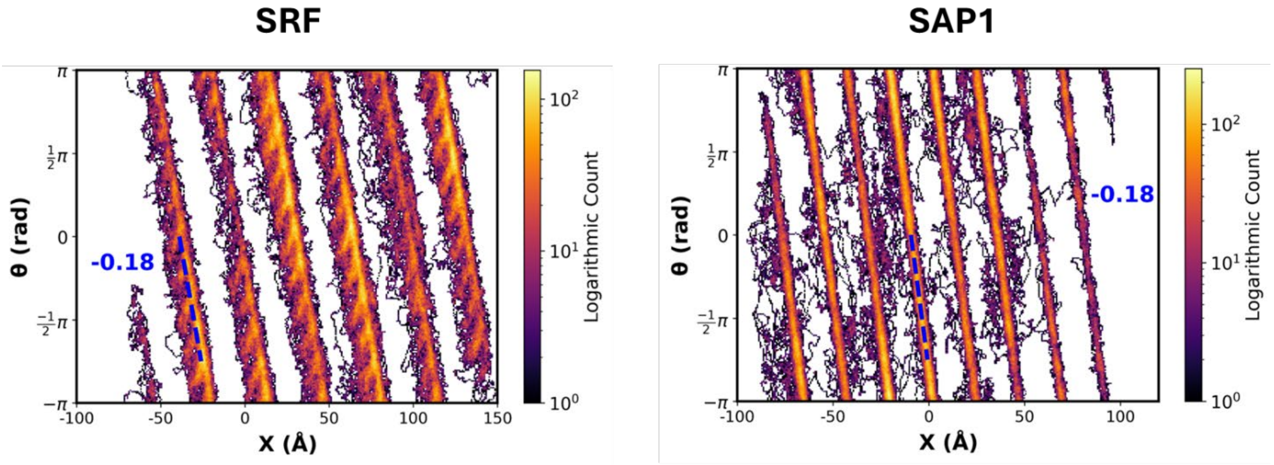

Figure S7. Heatmaps of protein diffusion trajectories in the [X,θ] space for SAP1 and SRF. The motion mechanism is analyzed by projecting five sampled protein trajectories onto the [X,θ] space at a salt concentration of 0.02M, where X represents the center of mass (COM) position of the diffusing protein along the DNA axis, and θ denotes the protein's rotational angle. Sliding motion is characterized by a coupling between rotation and translation, revealed by a linear relationship between θ and X with an approximate slope of $-0.18\frac{rad}{\text{Å}}$ (dashed blue line). Scattered points deviating from the slope lines indicate diffusion via hopping or alternatively, engagement in 3D diffusion. SAP1 was simulated with its tail included, but the center of mass (COM) was calculated excluding the tail.

References

1. Rogoulenko,E. and Levy,Y. (2024) Skipping events impose repeated binding attempts: profound kinetic implications of protein-DNA conformational changes. *Nucleic Acids Res*, **52**, 6763–6776.

2. Bigman,L.S., Greenblatt,H.M. and Levy,Y. (2021) What Are the Molecular Requirements for Protein Sliding along DNA? *J. Phys. Chem. B*, **125**, 3119–3131.
